# ProbABEL package for genome-wide association analysis of imputed data

**DOI:** 10.1186/1471-2105-11-134

**Published:** 2010-03-16

**Authors:** Yurii S Aulchenko, Maksim V Struchalin, Cornelia M van Duijn

**Affiliations:** 1 Department of Epidemiology, Erasmus MC, Postbus 2040, 3000 CA Rotterdam, The Netherlands; 2 Institute of Cytology and Genetics SD RAS, Novosibirsk, 630090, Russia

## Abstract

**Background:**

Over the last few years, genome-wide association (GWA) studies became a tool of choice for the identification of loci associated with complex traits. Currently, imputed single nucleotide polymorphisms (SNP) data are frequently used in GWA analyzes. Correct analysis of imputed data calls for the implementation of specific methods which take genotype imputation uncertainty into account.

**Results:**

We developed the ProbABEL software package for the analysis of genome-wide imputed SNP data and quantitative, binary, and time-till-event outcomes under linear, logistic, and Cox proportional hazards models, respectively. For quantitative traits, the package also implements a fast two-step mixed model-based score test for association in samples with differential relationships, facilitating analysis in family-based studies, studies performed in human genetically isolated populations and outbred animal populations.

**Conclusions:**

ProbABEL package provides fast efficient way to analyze imputed data in genome-wide context and will facilitate future identification of complex trait loci.

## Background

Genome-wide association (GWA) studies became the tool of choice for the identification of loci associated with complex traits. In GWA analyses, association between a trait of interest and genetic polymorphisms (usually single nucleotide polymorphisms, SNPs) is studied using thousands of people typed for hundreds of thousands of polymorphisms. Several hundred loci for dozens of complex human disease and quantitative traits have been discovered thus far using this method [1].

For any given genetic polymorphism, association can be studied using standard statistical analysis methodology, such as fixed and mixed effects models. However, because of the large number of tests to be performed and the quantity of data to be stored in GWA studies, computational throughput and effective data handling are essential features of statistical analysis software to be used in this context. A number of specialized software packages, such as PLINK[2], GenABEL[3], SNPTEST[4] and snpMatrix[5] were developed for the statistical analysis of GWA data. Most of these packages were designed, and are fit for, the analysis of directly typed SNPs. When directly typed markers are studied, genotype calling is performed with a high degree of confidence for the vast majority of markers, resulting in four possible genotypes ("AA", "AB", "BB", and missing). This allows representation of each individual genotype using two-bit coding and consequently effective storage of the genotype data in RAM [3].

Recently, novel statistical tools for genotype imputations [4,6-9] and experimental techniques for high-throughput sequencing were developed. Implementation of these methods usually results in estimates of the posterior probability distributions **P**_*g *_= (*P*_*AA*_, *P*_*AB*_, *P*_*BB*_) of the genotypes based on the available data. For many genomic loci, this distribution may be non-degenerate.

Several techniques can be applied to analysis of such "uncertain" data. The most simplistic approach would be to use the "best guess genotypes", that is to use the genotype with the highest posterior probability (*g *= max_*g *_*P*_*g*_) for analysis as if it were a directly typed markers. This approach is equivalent to replacing the estimated probability distribution with a degenerate one where a probability of one is assigned to the genotype with the maximal posterior probability. From standard statistical theory it is known, however, that such a procedure results in biased estimates of the effects. A correct analysis can be achieved using a maximum likelihood approach. Under this approach the likelihood can be computed using the total probability formula in which summation is performed over the genotypes, whose true values are not known, but whose posterior probabilities can be estimated given the data. This approach is computationally demanding, as it requires summation over the underlying probability distribution and numerical maximization of the likelihood function. Alternatively, a regression approach in which the posterior genotypic probabilities are used as predictors, can be applied. The main advantage of this approach is that well-established regression analysis methodology, algorithms, and code can be used in its implementation. Most currently available packages for GWA analysis can not be directly used in this manner, as they assume degenerate genotypic distributions and do not provide a facility for the storage and analysis of real-number predictors (posterior genotypic probabilities).

In this work, we describe the ProbABEL package, which was designed to perform genome-wide regression on posterior genotypic probabilities estimated using imputation software, such as MACH[6] or IMPUTE[4,9]. In addition to standard linear and logistic regression, which is widely applied to the analysis of quantitative and binary outcomes in population-based GWA studies, we also implemented a Cox proportional hazards model. For quantitative traits, we implemented a fast two-step mixed model-based score test for association testing in studies with a high degree of confounding induced by differential relationships between study subjects (e.g. family-based studies, studies of human genetically isolated populations, and studies in outbred animal populations).

## Implementation

Here, in the first few sub-sections, we will describe ProbABEL software, giving only the main outline of the underlying theory and with special emphasis on implementation and the options allowing to access specific analyzes within ProbABEL. In two last sub-sections, starting with the "Fixed effects model theory", we will give more in-depth review of the theory used by the package.

ProbABEL was implemented using code written in the C and C++ languages. The package consists of three executable files, used to perform linear, logistic, and Cox regressions, and a helper Perl script which facilitates the analysis of multiple chromosomes.

The package implements standard regression analysis methodology outlined in the section "Fixed effects model theory" and specific approximation to the mixed linear model described in the section "Two-step score test approximation to the mixed model". The key statistical tests performed by ProbABEL concern testing of the SNP effects. Here, we will describe the tests performed by ProbABEL using an example of linear regression; testing using other types of regression follows similar logic.

In linear regression, the expectation of the trait is described as

where **Y **is the vector of phenotypic values, **X**_*g *_is the design matrix containing data about predictors of interest (these involving SNP data), and **X**_*x *_is the design matrix containing other (nuisance) covariates. *β*_*g *_and *β*_*x *_are the vectors of corresponding fixed effects. The vector of phenotypes **Y **and the covariates matrix **X**_*x *_are provided in the phenotype file. The genotypic data are read from the genotype (dose or probability) files and are analyzed one SNP at a time.

Our interest lies in testing the (components of) *β*_*g *_. ProbABEL provides the estimates of the components of the vector *β*_*g *_and corresponding standard errors, and, in most cases, the test of the general hypothesis concerning the involvment of the SNP, obtained by comparison of the estimated model to the null model formulated as *β*_*g*,0 _= **0**, where **0 **is the vector of zeros.

Under the general genotypic model, **X**_*g *_is a matrix with the number of rows equal to the number of people under consideration and with two columns. Each row of the matrix contains the estimated probabilities that a person has genotype "AA" or "AB". Then, the vector of genotypic effects is described with two parameters: *β*_*g *_= (*β*_*AA*_, *β*_*AB*_). Thus formulated, the model allows for the estimation of a general genotypic two-degree of freedom model. Further, a number of sub-models can be formulated by setting restrictions on these parameters. The "dominant B allele" model is formalized as *β*_*AB *_= 0, "dominant A" (the same as "recessive B") as *β*_*AA *_= *β*_*AB*_, the additive model as 2 · *β*_*AB *_= *β*_*AA*_, and the over-dominant model as *β*_*AA *_= 0. Note that the additive model is equivalent to performing linear regression on the estimated dose of allele "A" defined as *P*_*AB *_+ 2 · *P*_*AA*_. The latter model is tested when the allelic dosage file is provided as the input for ProbABEL, while the full range of described models is tested if the estimated probability files (option "--ngpreds = 2") are supplied.

ProbABEL can also test for interaction between a specified covariate and the set of SNPs; for that alternative, the interaction covariate should be specified using the "--interaction N" option, where N corresponds to the number of the column of the design matrix **X**_*x*_, which contains that covariate. If this option is used, the expectation of the trait is defined as

where **W **is a diagonal matrix, whose diagonal elements are formed by substituting the interaction covariate to the matrix and *β*_*gxe *_is the vector of interaction regression coefficients.

### Analysis of population-based data

If the study subjects can be assumed to be genetically "independent", in the sense that they come from the general outbred population without a marked degree of stratification and that cryptic relatedness is absent, the data can be effectively analyzed using standard linear fixed effects regression methodology, as described in section "Fixed effects model theory". The (small) effects of confounding can be corrected posterior to analysis using the genomic control [10] procedure. If a marked degree of stratification is present, such methods as structured association analysis and EIGENSTRAT [11] can be combined with the standard methods.

Using standard methods, the estimates of the parameters can be obtained using the standard formula 1 (see "Fixed effects model theory" below), which provides maximum likelihood estimates if (*X*^*T*^*X*)^-1 ^exists. The latter condition is fulfilled for virtually all analyses; practically, exceptions may occur for SNPs with very low minor allele frequencies or poor quality imputations.

The standard errors are computed as square roots of the diagonal elements of the parameter estimates' variance-covariance matrix. This matrix is computed using one of three different methods: the standard method, with residual variance estimated under the alternative (formula 2, see "Fixed effects model theory" below) or null hypothesis concerning SNPs (option "--score"), or using a "sandwich" estimator (formula 5, see "Fixed effects model theory"), resulting in robust standard errors (option "--robust"). The value of the global likelihood ratio test statistic, testing the joint significance of all terms involving SNP, is computed using the formula 3 (see "Fixed effects model theory"). In this test, the null model is formulated as *β*_*g*,0 _= **0**, where **0 **is the vector of zeros. If an interaction term is present, that is also set to zero under the null: *β*_*gxe*,0 _= **0**. The likelihoods involved are computed using the formula 4 (see "Fixed effects model theory") with the values of the parameters fixed at the point of the maximum likelihood estimate obtained with 1 (see "Fixed effects model theory").

### Analysis of data on subjects with differential relationships

In the case of a study involving subjects with markedly differential relationships (family-based designs, studies of human genetically isolated populations, studies in outbred animal populations), a mixed model approach may be used, in which a random effect ("heritability") accounts for similarities between the phenotypes of study subjects [12]. However, the estimation of the full mixed model using either maximum likelihood or the restricted maximum likelihood approach is computationally demanding, if not unfeasible, within the framework of GWAS [13], and therefore a two-step mixed model-based approach [13-15] is utilized in ProbABEL.

In this approach, the mixed model containing all terms but those involving SNP is first estimated by maximizing the likelihood function provided by the expression 7 (see section "Two-step score test approximation to the mixed model" for details). These estimates are then used in the second step to compute estimates of the SNP effects (formula 8 of "Two-step score test approximation to the mixed model") and the variance-covariance matrix of these estimates (formula 10, see "Two-step score test approximation to the mixed model"). These values can be used to perform a score test for association. The second step of a mixed-model based score test for association is available in ProbABEL using option "--mmscore IVFile", where IVFile is the name of a file containing the inverse of the variance-covariance matrix ( of formulas 8 and 10, see "Two-step score test approximation to the mixed model") evaluated at the point of the maximum likelihood estimates obtained in step one. The phenotypes analyzed in the second step are residuals (as specified by the formula 9, see "Two-step score test approximation to the mixed model") obtained by subtracting the trait values expected under the mixed model-based estimates of the fixed effects from the original trait values.

Step one of the regression procedure can be performed using our GenABEL software [3]. This software performs genomic data based estimation of the kinship matrix as described in section "Estimation of genomic kinship matrix" using the ibs (...,weight="freq") function, and performs maximum likelihood estimation of the step-one mixed model using the polygenic() function. The resulting object contains the inverse variance-covariance matrix (object$InvSigma), which can be saved as a text file and used in ProbABEL analysis. The residuals to be used as trait values in step two of the analysis can be accessed through object$residualY.

### Input and output

The input consists of a phenotypic data file and a set of files describing the imputed genotypic data. The phenotypic file provides data on the outcome of interest and any additional covariates to be included in the analysis. The genotypic data files, at present, utilize the MACH imputation software output format. Minimally, a file with estimated probability distributions ("mlprob") or allelic dosages ("mldose") and the "mlinfo" file containing information about allele coding and overall imputation quality should be provided. Optionally, a map file in HapMap format, containing chromosome and location information, may be supplied. Information contained in the latter two files is not used in analysis, but is forwarded directly to the output. If the mixed-model based score test for association in related individuals is to be computed, a file containing the inverse matrix of variances and covariances between the phenotypes of study individuals should be supplied as a part of the input. The output of the program consists of one line for each SNP tested, containing information about the SNP supplied as part of the input, as well as the results from analysis (estimates of the coefficients of regression, standard errors of the coefficients, and test statistic values).

### Fixed effects model theory

Most of the fixed effects model theory outlined here is standard and can be found in textbooks, such as "Generalized, Linear, and Mixed Models" [16]. Specific references are provided when this is not the case.

#### Linear regression assuming normal distribution

Standard linear regression theory is used to estimate coefficients of regression and their standard errors. We assume linear model with expectation

and variance-covariance matrix

where **Y **is the vector of phenotypes of interest, **X **is design matrix, *β *is the vector of regression parameters, *σ*^2 ^is variance and **I **is identity matrix.

The maximum likelihood estimates (MLEs) for the regression parameters is given by(1)

and MLE of the residual variance is

where *N *is the number of observations and *r*_*X *_is rank of **X **(number of columns of the design matrix).

The variance-covariance matrix for the parameter estimates under alternative hypothesis can be computed as(2)

For the *j*-the element (*j*) of the vector of estimates the standard error under alternative hypothesis is given by the square root of the corresponding diagonal element of the above matrix, **var**__(*jj*), and the Wald test can be computed with

which asymptotically follows the *χ*^2 ^distribution with one degree of freedom under the null hypothesis. When testing significance for more than one parameter simultaneously, several alternatives are available. Let us partition the vector of parameters into two components, *β *= (*β*_*g*_, *β*_*x*_), and our interest is testing the parameters contained in *β*_*g *_(SNP effects), while *β*_*x *_(e.g. effects of sex, age, etc.) are considered nuisance parameters. Let us define the vector of the parameters of interest which are fixed to certain values under the null hypothesis as *β*_*g*,0 _(usually, *β*_*g*,0 _= **0**, vector of zeros).

The likelihood ratio test can be obtained with(3)

which under the null hypothesis is asymptotically distributed as *χ*^2^with number of degrees of freedom equal to the number of parameters specified by *β*_*g *_. Assuming the normal distribution, the log-likelihood of a model specified by the vector of parameters *β *and residual variance *σ*^2 ^can be computed as(4)

Secondly, the Wald test can be used; for that the inverse variance-covariance matrix of _*g *_should be computed as

where  correspond to sub-matrices of the inverse of the variance-covariance matrix of , involving either only covariances between the parameters of interest (*g, g*), only the nuisance parameters (*x, x*) or between the parameters of interest and nuisance parameters, (*x, g*), (*g, x*).

The Wald test statistics is then computed as

which asymptotically follows the *χ*^2 ^distribution with the number of degrees of freedom equal to the number of parameters specified by *β*_*g *_. The Wald test generally is computationally easier than the LRT, because it avoids estimation of the model specified by the parameter's vector (*β*_*g*,0_,_*x*_).

Lastly, similar to the Wald test, the score test can be performed by use of  instead of **var**__.

#### Logistic regression

For logistic regression, the procedure to obtain parameters estimates, their variance-covariance matrix, and tests are similar to these outlined above with several modifications.

The expectation of the binary trait is defined as expected probability of the event as defined by the logistic function

The estimates of the parameters are obtained not in one step, as is the case of the linear model, but using iterative procedure (iteratively re-weighted least squares). This procedure is not described here for the sake of brevity.

The log-likelihood of the data is computed using binomial probability formula:

where *log*_*e*_*π *is a vector obtained by taking the natural logarithm of every value contained in the vector *π*.

#### Robust variance-covariance matrix of parameter estimates

For computations of robust variance-covariance matrix we use White's sandwich estimator [17,18], which is equivalent to the "HC0" estimator described by Zeilers and Lumley in "sandwich" package for R.

For linear model, the variance-covariance matrix of parameter estimates is computed using formula

where **R **is a diagonal matrix containing squares of residuals of **Y**. The same formula may be used for "standard" analysis, in which case the elements of the **R **matrix are constant, namely mean residual sum of squares (the estimate of residual variance, ).

Similar to that, the robust matrix is computed for logistic regression with

where **W **is the diagonal matrix of "weights" used in logistic regression.

#### Cox proportional hazards model

The implementation of the Cox proportional hazard model used in ProbABEL is entirely based on the code of R library survival developed by Thomas Lumley (function coxfit2), and is therefore not described here.

### Two-step score test approximation to the mixed model

The framework for analysis of data containing differential relationships follows the two-step logic developed in the works of Aulchenko et al. [13] and Chen and Abecasis [14]. General analysis model is a linear mixed model which defines the expectation of the trait as

identical to that defined for linear model. To account for possible correlations between the phenotypes of study subjects the variance-covariance matrix is defined to be proportional to the linear combination of the identity matrix **I **and the relationship matrix **Φ**:

where *h*^2 ^is the heritability of the trait. The relationship matrix **Φ **is twice the matrix containing the coefficients of kinship between all pairs of individuals under consideration; its estimation is discussed in a separate section "Estimation of genomic kinship matrix".

Estimation of thus defined model is possible by numerical maximization of the likelihood function, however, the estimation of such model for large data sets is not computationally feasible for hundreds of thousands to millions of SNPs tested in the context of GWAS, as we have demonstrated previously [13].

#### Two-step score test for association

A two-step score test approach is therefore used to decrease the computational burden. Let us re-write the expectation of the trait by splitting the design matrix in two parts, the "base" part **X**_*x*_, which includes all terms not changing across all SNP models fit in GWAS (e.g. effects of sex, age, etc.), and the part including SNP information, **X**_*g*_:

Note that the latter design matrix may include not only the main SNP effect, but e.g. SNP by environment interaction terms.

At the first step, linear mixed model not including SNP effects

is fitted. The maximum likelihood estimates (MLEs) of the model parameters (regression coefficients for the fixed effects _*x*_, the residual variance  and the heritability ) can be obtained by numerical maximization of the likelihood function(7)

where  is the inverse and  is the determinant of the variance-covariance matrix.

At the second step, the estimates of the fixed effects of the terms involving SNP are obtained with(8)

where  is the variance-covariance matrix at the point of the MLE estimates of  and  and(9)

is the vector of residuals obtained from the base regression model. Under the null model, the inverse variance-covariance matrix of the parameter's estimates is defined as(10)

Thus the score test for joint significance of the terms involving SNP can be obtained with

where *β*_*g*,0 _are the values of parameters fixed under the null model. This test statistics under the null hypothesis asymptotically follows the *χ*^2 ^distribution with the number of degrees of freedom equal to the number of parameters tested. The significance of an individual *j*-the elements of the vector _*g *_can be tested with

where  is square of the *j*-th element of the vector of estimates _*g*_, and  corresponds to the *j*-th diagonal element of . This statistics asymptotically follows .

#### Estimation of genomic kinship matrix

The relationship matrix **Φ **used in estimation of the linear mixed model is twice the matrix containing the coefficients of kinship between all pairs of individuals under consideration. This coefficient is defined as the probability that two gametes randomly sampled from each member of the pair are identical-by-descent (IBD), that is they are copies of exactly the same ancestral allele. The expectation of kinship can be estimated from pedigree data using standard methods, for example the kinship for two outbred sibs is 1/4, for grandchild-grandparent is 1/8, etc. However, in many situations, pedigree information may be absent, incomplete, or not reliable. Moreover, the estimates obtained using pedigree data reflect the expectation of kinship, while the true realization of kinship may vary around this expectation. In presence of genomic data it may therefore be desirable to estimate the kinship coefficient from these, and not from pedigree. It can be demonstrated that unbiased and positive semi-definite estimator of the kinship matrix [19] can be obtained by computing the kinship coefficients between individuals *i *and *j *with

where *L *is the number of loci, *p*_*l *_is the allelic frequency at *l*-th locus and *g*_*l, j *_is the genotype of *j*-th person at the *l*-th locus, coded as 0, 1/2, and 1, corresponding to the homozygous, heterozygous, and other type of homozygous genotype [11,15,19]. The frequency is computed for the allele which, when homozygous, corresponds to the genotype coded as "1'.

## Results

To ensure the statistical correctness of the two-step procedure, we performed a small-scale simulation study. We used real data from the Erasmus Rucphen Family (ERF) study [20]. In simulations, we used genotypic data from 2,313 people who had high-density SNP genotyping data. The trait was simulated as a sum of four independent effects: two fixed effects explaining 10 and 5% of the total trait variance, a polygenic effect, and a residual random effect. The residual random effect was assumed to be distributed normally with mean zero and variance fixed at the value that explained 59.5% of total variance. To simulate the polygenic effect, similar to our previous work [15], we selected 200 random SNPs, and assigned these SNPs with fixed effects such that, in total, these SNPs explained 25.5% of total variance. Thus, the heritability of the trait when adjusted for the fixed effects was 30%.

The SNPs mimicking the polygenic effect were selected randomly from all autosomes but the second. To estimate type 1 error of the two-step procedure, we studied association of the trait with the second chromosome SNPs using real imputed data. Only SNPs with estimated minor allele frequencies greater than 1% were used in analysis (212,691 SNPs in total). We compared type 1 error rates for four different models: a linear model ignoring the relatedness structure (using both a standard and a robust covariance matrix) and our 2-step mixed-model based score test. For the latter, we adjusted for two fixed effect covariates in the first step (polygenic) analysis.

The results of these tests are summarized in Table 1. It is easy to see that when relationships between study individuals are not taken into account, the distribution of the test statistic is inflated, regardless of whether a robust or standard covariance matrix is used. In our previous work, we demonstrated that this inflation grows with increasing trait heritability, with more close relatives present in the sample [15] and with increasing sample size and can reach very high values. On the contrary, when two-step approximation to the mixed model is used ("Linear, mmscore" row of Table 1), the test statistic shows very good agreement to the  distribution expected under the null.

**Table 1:** Mean values of the test statistics (Wald for Linear, score for mmscore), genomic control *λ *(median test statistic over 0.455), and type 1 error at different *α *for different models.

			α		
			
Model	Mean(*T*^2^)	*λ*	0.05	0.01	0.001
Linear	1.206	1.224	0.073	0.018	0.0027
Linear, robust	1.210	1.228	0.073	0.018	0.0028
Linear, mmscore	0.984	1.007	0.047	0.009	0.0011

Tests were performed using a trait dependent on two covariates and with (adjusted) heritability of 30%. Only SNPs with estimated minor allele frequency greater than 0.01 (*n *= 212, 691) used. Linear: standard linear model; Linear, robust: linear models using with standard errors; Linear, mmscore: two-step approximation to mixed model, fixed effects included in step 1 of analysis.

Next, we measured CPU time required for particular ProbABEL analyses. To do this, we selected 500, 1000, and 1500 people from 2,313 genotyped individuals and measured the speed of different types of analysis using chromosome 2 imputed data on 220,833 SNPs. All analyses were ran on a Sun Fire X4640 server with an Intel Xeon CPU 5160 (3.00 GHz). Results are present in Table 2. ¿From this table, it is clear that all population-based analyzes (these not involving the --mmscore option) scale roughly linearly with the number of people. Use of the --robust option increases the running time by only a small fraction. Based on these data, one would expect that a GWA analysis involving, for example, 2.5 millions SNPs imputed on HapMap2 release 22 in 1,500 individuals would take 1/2 hour for linear, 2 hours for logistic and 1 1/2 hours for Cox proportional hazards models.

**Table 2:** Time for analysis of chromosome 2 imputed data (220,833 SNPs).

Model	Option	No. people	CPU time
Linear	-	500	0 m 43 s
		1000	1 m 23 s
		1500	2 m 10 s

Linear	--robust	500	0 m 50 s
		1000	1 m 43 s
		1500	2 m 35 s

Linear	--mmscore	500	16 m 18 s
		1000	92 m 45 s
		1500	231 m 49 s

Logistic	-	500	3 m 20 s
		1000	6 m 38 s
		1500	10 m 8 s

Logistic	--robust	500	3 m 25 s
		1000	6 m 53 s
		1500	10 m 29 s

Cox PH	-	500	2 m 18 s
		1000	4 m 30 s
		1500	6 m 43 s

In all analyzes, 2 covariates were included in the model.

Use of the --mmscore option to adjust for relationships between study subjects, however, induces a non-linear relationship between the number of study subjects and analysis time: while the time to analyze 500 people is 16 minutes, the time for analysis of 1500 people is *≈ *14 times longer. The time for a GWA with 1,500 people and 2.5 millions imputed SNPs is, therefore, estimated to be *≈ *43 hours.

## Discussion

Imputed SNP data are conventionally used for the analysis of GWA data; correct use of imputed data allows for higher power and location accuracy [21,22]. However, correct analysis of imputed data needs to account for the uncertainty surrounding estimated genotypic probability distributions. This can be done using approaches based on either likelihood or regression on estimated probabilities, as outlined in the "Background" and "Implementation". A number of software packages are available for such analyses. SNPTEST implements a score test based on missing data likelihood [4] allowing for the study of both quantitative and binary outcomes. MACH2QTL and MACH2DAT implement regression models on estimated probabilities for quantitative and binary traits, respectively, in a manner similar to ProbABEL. ProbABEL extends the functionality available in these packages by allowing analysis under the Cox proportional hazards model. Further, while SNPTEST allows for testing interaction of a covariate with SNPs studied, it does not provide the value of the global significance test. Finally, ProbABEL is the only package that implements specific mixed-model based procedures for the study of association in samples with differential relationships, facilitating analysis in family-based studies, studies performed in human genetically isolated populations, and outbred animal populations.

In theory, the mixed model we have described can also be used to correct for population stratification in a study where a number of (population-based and family based) samples come from differentiated genetic populations [12,19]. However, given the different genetic and potentially different environmental compositions of such differentiated populations, similar heritabilities can not be assumed in all study populations. We speculate that, in practice, one should combine population-specific (fixed or mixed-model) approaches with structured association or similar methods. For example, one could identify sets of individuals coming from divergent genetic populations using either prior information or analysis of the principal components of the genomic kinship matrix [11]; perform standard analysis in population-based sets and mixed-model analysis in family based sets (or those exhibiting substantial cryptic relatedness), as described here; and finally combine the results using meta-analysis. The best strategy to analyze such complex studies is to be addressed elsewhere in more details.

The two-step mixed model-based score test implemented in ProbABEL is an extension of the family-based association score test suggested by Chen and Abecasis [14], and is similar in its logic to the GRAMMAR and GRAMMAR-GC tests described by Aulchenko et al. [13,15]. In the test procedure, the model is split into two parts (see the equation 6 in "Two-step score test approximation to the mixed model"), the first of which contains the effects of nuisance parameters, including random genetic effects, and the second includes the parameters of interest (SNP effects and SNP-interacting covariates). Estimation in the second step is performed based on the estimates obtained from fitting the first part. Strictly speaking, the test defined in this manner is correct if the distributions of covariates in the first and the second parts of the model are independent conditional on the estimated phenotypic variance-covariance matrix. This assumption is most likely to be true when the covariates included in the base model are environmental ones, and thus are not expected to exhibit conditional correlation with SNPs. However, when endogenous risk factors, such as body mass index, are included as the covariates in the base model, some SNPs are expected to exhibit covariance with this covariate. In such situations, the covariate should be included in the second step analysis. This, however, may violate the assumptions of the score test if the covariate explains a large proportion of trait variance. In such situation we expect that the test will become conservative and may be less powerful compared to the classical maximum likelihood analysis.

At present, GWA analysis of millions of imputed SNPs using the --mmscore option in ProbABEL takes a few days for samples of a few thousands of people. However, the relationship between CPU time and the number of subjects is not linear; as the number of subjects reaches 5,000 or more, the mixed-model based analysis will take too much time (weeks to months) when using a single CPU. A straightforward approach to solve this problem would be to use parallel computations. Still, the non-linear dependency of computational time on the number of subjects may become a major analysis bottleneck with larger and larger studies becoming available.

Other software packages which implement similar mixed-model functionality and are suitable for GWA analyses are MERLIN [23] and QxPak [24]. In particular, MERLIN implements the two-step score test [14], which is equivalent to our test in the absence of covariates. QxPak is a flexible tool for mixed modeling of quantitative traits, which implements classical full Maximum Likelihood and Restricted Maximum Likelihood estimation procedures. Neither MERLIN nor QxPak, however, allow for analyses of imputed data in the form of regression onto estimated genotype probabilities. Both packages assume that pedigree structure is known, and estimate kinship based on that.

On the contrary, the input required by ProbABEL consists of the inverse matrix of estimated variances and covariances between the phenotypes of study individuals. This matrix can be obtained in a number of different ways; our standard approach is to estimate it using GenABEL's polygenic() function based on kinship estimated from genomic data, as computed with the ibs(..., weight="freq") function.

However, it is possible and straightforward to use kinship estimated from pedigree data as well (using, e.g., "kinship" library of R) in the polygenic() procedure. The latter approach is preferable in a study where no genome-wide data is available for estimation of genomic kinship (such as a candidate gene or region study).

Presently, there is no package (including ProbABEL), which allows for genome-wide association analysis of binary traits or time-till-event outcomes under a mixed model or an approximation to a mixed model accounting for relatedness, and providing the correct estimates of Odds or Hazards Ratios. With the growing number of GWA scans performed in families and genetically isolated populations, this gap needs to be filled.

For population-based analyses using fixed effects models, ProbABEL computes Maximum Likelihood estimates of the parameters and the standard errors under the alternative hypothesis, allowing a Wald test for every parameter under consideration. The global SNP significance test is implemented using the Likelihood Ratio Test. Theoretically, the Wald test can be used for the same purpose, thereby avoiding the need to re-estimate the null model with respect to each SNP. However, in GWAS with imputed data, where full information is available for all SNPs, the null model estimation needs to be performed only once, and can then used for testing all SNPs. Thus the overhead related to re-estimation of the null model is minimal, and, for that reason, we did not implement the global SNP significance Wald test.

We should emphasise that, in general, the ProbABEL software can be used to do massive regression analyzes using any type of real-type outcomes and predictors. As such, ProbABEL is not restricted to SNP, or even, more generally, to genetic analyzes and can be used for any analyzes requiring regression of a dependent variable on a very large number of independent variables in turn. For example, ProbABEL may be use to perform association testing among traits and Copy Number Polymorphisms [25].

The practical applicability of ProbABEL for the analysis of GWAS is confirmed by the fact that the early versions of the package were successfully used for analysis of multiple data sets, including already published genome-wide analyzes of such various traits as height [26,27], gout [28], waist circumference [29], smoking initiation [30], and others.

## Conclusions

We developed the ProbABEL software package, which facilitates fast genome-wide association analysis of imputed data under linear, logistic and Cox proportional hazards models. For quantitative traits, the package also implements a two-step mixed model-based score test for association in samples with differential relationship, facilitating analysis in family-based studies, studies performed in human genetically isolated populations, and outbred animal populations.

## Availability and requirements

**Project name: **ProbABEL

**Project home page: **http://mga.bionet.nsc.ru/~yurii/ABEL/ (source code and binaries for various platforms), http://r-forge.r-project.org/projects/genabel/ (project development page)

**Operating system(s): **source code was successfully compiled and used on Windows, Mac OS X, Linux, SUN Solaris

**Programming language: **C, C++, Perl

**Other requirements: **make

**License: **GNU GPL

**Any restrictions to use by non-academics: **None

## Authors' contributions

YSA developed the original idea, methodology, and code for the fixed effects part. MVS contributed the code for the interaction testing and two-step mixed-model based procedures. CvD provided ERF study data. All co-authors contributed to writing of the manuscript.
